# Level of knowledge among health care providers on preparation of injectable artesunate for treatment of severe malaria in public health facilities in Tanzania

**DOI:** 10.1186/s13104-019-4257-5

**Published:** 2019-04-11

**Authors:** Wigilya P. Mikomangwa, Calvin Kaaya, Manase Kilonzi, Hamu Mlyuka, Alphonce Ignace Marealle, Ritah Mutagonda

**Affiliations:** Muhimbili University of Health & Allied Sciences, Dar-es-Salaam, Tanzania

**Keywords:** Severe malaria, Injectable artesunate, Health care providers

## Abstract

**Objective:**

In Tanzania, seminars and training on the preparation and administration of injectable artesunate were given to health care providers (HCP) during its introduction in 2013. Published evidence on knowledge of its preparation among HCP in public health facilities is scarce. The study determined level of knowledge of health care providers (HCP) in public health facilities on the preparation of injectable artesunate for severe malaria.

**Results:**

Most (82.5%) of HCP had low knowledge on preparation of injectable artesunate; 78.8% of HCP did not know what to do if the mixture do not mix-up correctly, 73.7% did not know when to discard the preparation if not used and half (50.1%) of them knew how to correctly calculate the dose based on body weight.

## Introduction

Malaria is one of the serious diseases in tropical areas affecting about 40% of the world population [1]. Each year malaria cases are estimated to be between 300 and 500 million and severe malaria is estimated to kill about 1 million people annually majority being children [1]. Sub-Saharan Africa has been reported to contribute 90% of malaria cases worldwide [1]. In Tanzania malaria accounts for national disease burden about 30% and most of the malaria infections attributed by *Plasmodium falciparum* [2, 3].

In Tanzania mainland, injectable artesunate is the first drug of choice while artemether injection is the alternative choice in treatment of severe malaria in the general population and quinine injection is the first line in treatment of severe malaria in pregnant women [3]. Injectable artesunate gains advantage over other antimalarials in treating severe malaria due to its tolerable side effects and high parasitic clearance. It is associated with low mortality in both children and adults with fewer side effects compared to quinine [3–5]. Injectable artesunate formulation is a sterile powder containing artesunic acid which must be in the range 90–110% of the artesunate stated on the label [6]. The vial of artesunic acid comes with a 1 ml ampoule of diluents sodium bicarbonate solution and one ampoule of normal saline. The powder of artesunic acid should be mixed with sodium bicarbonate to form hemisuccinate ester of artemisinin which is therapeutically active [7]. The reconstituted solution should be discarded if precipitate or appears cloudy for more than 2 min [8]. The clear solution should be diluted using normal saline (0.9% sodium chloride) or 5% dextrose solution to make concentration suitable for either IV or IM administration [9]. Artesunate is unstable in aqueous solutions therefore must be used within 1 h after preparation and not to be stored in refrigerator and the unused solution should be discarded [9].

Since the introduction of injectable artesunate in Tanzania in 2013 sufficient published reports on the level of knowledge of HCP on preparation of injectable artesunate in public health facilities is lacking. Therefore this study aimed at assessing the level of knowledge of HCP on the preparation of injectable artesunate for the treatment of severe malaria in public health facilities in Dar Es Salaam Region.

## Main text

### Methodology

#### Study design and study area

A cross-sectional study design was employed to assess the level of knowledge among HCP on preparation of injectable artesunate in public health facilities in Ilala, Kigamboni, Kinondoni, Temeke, and Ubungo municipals in Dar-es Salaam region.

#### Study population and sample size

Since no previous studies resembling to this, the prevalence of low knowledge on preparation of artesunate injection for management of severe malaria among HCP in public health facilities was set at 50%. Using formula for calculating sample size for cross section study [10] with 95% confidence interval, 5% margin of error and non-response rate of 3% the sample size was 396 HCP.

#### Sampling technique

The study involved cluster sampling by which the list of all health facilities (health centers, district hospitals and referral hospitals) were obtained from Regional Medical Officer’s office. Health facilities were randomly selected using ballot method to include 5 hospitals (3 district and 2 regional referral hospitals), 8 health centers and 12 dispensaries. Each HCP who met the criteria (nurses, clinician and pharmaceutical personnel) from the selected facilities were sampled using consecutive sampling technique.

#### Data collection

Pretested and validated questionnaire were used to assess knowledge of HCP after obtaining their consent. Questionnaire contained two sections which were socio-demographic information and knowledge on preparation of injectable artesunate with subsections on availability and preference of drug for treatment of severe malaria.

#### Data analysis

Descriptive statistics was used to summarize the demographic characteristics, cadre, work experience and level of health facility. Comparison of categorical data such as knowledge, qualification level, working experience, and working profession was done by Chi square, significance level p value < 0.05 was considered significant using SPSS version 20 software (IBM Corp. Released 2011. IBM SPSS Statistics for Windows, Version 20.0. Armonk, NY: IBM Corp). Bloom’s cut off points were used to grade the level of knowledge of health care workers as high (80–100%), moderate (60–79%) or low (0–59%) knowledge when the scores are 5–6, 3–4 and 0–2 respectively.

### Results

The study was conducted from January to March 2016 by which 377 HCP responded and 25 health care facilities were involved in the study. The mean age of respondents was 36.5 ± 9.5 years old, majority (69%) being female. Those with diploma qualification were half (53.4%) of the total participants. Majority (63.1%) of the participants were nurses with more than 4 years of experience from dispensaries (59.4%). Almost (93.1%) all HCP did not attend any training concerning preparation of injectable artesunate. Twenty-four (96.3%) health facilities had reference materials such as posters and standard operating procedures for preparation of injectable artesunate (Table 1). Injectable artesunate was available in 100%, 86% and 93% of hospitals, health centres and dispensaries respectively. Most (69.8%) of HCP preferred artesunate injection over quinine (27.5%) and artemether (4.5%) injection for management of severe malaria.

**Table 1:** Association between level of knowledge and characteristics of participants (n = 377)

Variables	n (%)	Level of knowledge		
		Not knowledgeable	Knowledgeable	P-value
Age group				
20–29	118 (31.3)	98 (83.1)	20 (16.9)	
30–39	116 (30.8)	100 (86.2)	16 (13.8)	0.386
40–49	99 (26.3)	80 (80.8)	19 (19.2)	
50+	44 (11.7)	33 (75.0)	11 (25.0)	
Sex				
Male	117 (31.0)	89 (76.1)	28 (23.9)	*0.028*
Female	260 (69.0)	222 (85.4)	38 (14.6)	
Qualification				
Certificate	101 (26.8)	85 (84.2)	16 (15.8)	
Diploma	200 (53.1)	163 (81.5)	37 (18.5)	0.844
Degree	76 (20.2)	63 (82.9)	13 (17.1)	
Profession				
Nurse	238 (63.1)	207 (87.0)	31 (13.0)	
Pharmaceutical personnel	33 (8.8)	28 (84.8)	5 (15.2)	*0.002*
Clinician	106 (28.1)	76 (71.7)	30 (28.3)	
Distribution of HCP in health facility				
Hospital	73 (19.4)	54 (74.0)	19 (26.0)	
Health Center	80 (21.2)	64 (80.0)	16 (20.0)	*0.047*
Dispensary	224 (59.4)	193 (86.2)	31 (13.8)	
Work experience, years				
< 1	43 (11.4)	36 (83.7)	7 (16.3)	
1–2	49 (13.0)	41 (83.7)	8 (16.3)	0.861
3–4	56 (14.9)	48 (85.7)	8 (14.3)	
> 4	229 (60.7)	186 (81.2)	43 (18.8)	
Reported availability of reference materials				
Yes	363 (96.3)	300 (82.6)	63 (17.4)	0.456
No	14 (3.7)	11 (78.6)	3 (21.4)	
HCP attendance to training				
Yes	26 (6.9)	16 (61.5)	10 (38.5)	*0.007*
No	351 (93.1)	295 (84.0)	56 (16.0)	

**Fig. 1:**
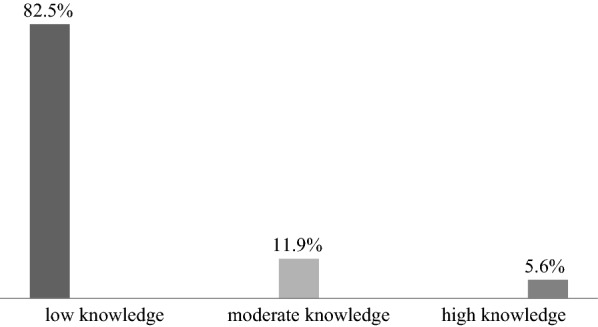
Level of knowledge of HCP regarding preparation of injectable artesunate (n = 377)

Majority (82.5%) of health care professionals had low knowledge and few (5.6%) had high knowledge on preparation and of injectable artesunate (Fig. 1). Nurses had significantly low level of knowledge compared to other professionals (p-value = 0.002) and HCP in dispensaries had marginally lower knowledge than those in health centers and hospitals (p-value = 0.047). Health care providers who did not attend any training (84.0%) for preparation of injectable artesunate had significantly low knowledge (p-value = 0.007) as shown in Table 1.

Of the 377 study participants, 55.4% responded correctly on the step by step procedure to follow when preparing injectable artesunate. However, majority did not know when to discard the preparation if the mixture do not mix-up properly (78.8%) or not used immediately after preparation (73.7%). Half of HCP did not know how to correctly calculate dose injectable artesunate based on body weight (Table 2).

**Table 2:** Frequency and percentage distribution table on the scores of responses of HCP on specific issues during preparation of injectable artesunate (n = 377)

Variables	Frequency	Percent (%)
The flow pattern to follow when mixing artesunate		
Incorrect	168	44.6
Correct	209	55.4
The step taken when the mixture did not mix up correctly		
Incorrect	297	78.8
Correct	80	21.2
When to discard the preparation if not used		
Incorrect	278	73.7
Correct	99	26.3
Difference in the preparation of IM and IV artesunate		
Incorrect	173	45.9
Correct	204	54.1
Difference in dose according to body weight (n = 307)		
Incorrect	20	6.5
Correct	287	93.5
Dose calculation based on body weight of patient (n = 355)		
Incorrect	178	50.1
Correct	177	49.9

### Discussion

This study reports the level of knowledge on the preparation of injectable artesunate for treatment of severe malaria among public health care providers in all municipalities of Dar-es-Salaam region. Majority of HCP had low knowledge (82.5%) on how injectable artesunate was prepared and only few had moderate (11.9%) to high knowledge (5.6%) despite of its high availability in health care facilities and preference by HCP.

The lack of knowledge of these HCP could be due to the reason that, majority (93.1%) of them reported to have not attended any training (seminar or workshop) which could be due to the lack of sufficient financial resources to facilitate training of all HCP. It could also be possible that, the few HCP who attended the training did not transfer the knowledge to their colleagues [11].

In general practice, nurses are the ones who prepare and administer injectable artesunate, but these nurses had significantly low knowledge on preparation of this medicine. This was also observed in Turkish study which found low knowledge among nurses in administering IM medicines at ventrogluteal site [11]. It has been reported that knowledge on drug dosage and preparation are essential in reducing errors [12]. Since other HCP were more knowledgeable than nurses, it could indicate lack of interprofession collaboration within studied health facilities [13, 14].

Given that almost all (96.3%) health facilities had reference materials and posters to guide preparation of injectable artesunate, the HCP could be relying on these references for preparing the injectable artesunate. This is in hand with the study conducted in Italy which reported the usefulness of posters, protocols and brochures in reducing medication errors for IV drugs administration [15]. Despite the usefulness of these reference materials, there is high chance of errors due to neglecting some important issues that could be emphasized during training. This could be reflected by the fact that almost half the HCP knew the flow pattern to follow when preparing artesunate injection and the difference between the IM and IV artesunate preparation but could not response correctly as what to do when the mixture do not mix up, when to discard the prepared solution or the correct dose by body weight. The challenges in preparation of injectable medicines by nurses have been reported in a Brazilian study by which incorrect dilution was identified as one of source of errors [16].

Since the general level of knowledge among HCP is very low, this may highlight the need of reevaluating the mode of delivery of training on preparation of injectable artesunate and the need of continue professional development as well as use of online platforms in delivering education which can reach many people within a short time. Posters and reference materials should not replace training but rather supplement the knowledge gained by attending seminars and workshops.

### Conclusion

The level of knowledge of among health care providers on preparation of injectable artesunate in public health facilities was low.

## Limitations

This was a cross-sectional study that determined only the level of knowledge on preparation of injectable artesunate but did not determine the actual practice in preparing the injectable artesunate. Therefore the results on the level of knowledge cannot be translated into the actual practice.

## Abbreviations

HCP: health care providers
IV: intravenous injection
IM: intramuscular injection

## References

[1] UNICEF. The global malaria burden malaria prevention and treatment. 2000;18:16.

[2] Mboera LEG, Makundi EA, Kitua AY (2007). Uncertainty in malaria control in Tanzania: crossroads and challenges for future interventions. Am J Trop Med Hyg.

[3] Ministry of Health and Social Welfare (2017). Standard treatment guidelines & national essential medicines list, Tanzania mainland G.

[4] Sinclair D, Donegan S, Dg L (2011). Artesunate versus quinine for treating severe malaria (Review). Health San Fr..

[5] SEAQUAMAT (2005). Artesunate versus quinine for treatment of severe falciparum malaria : a randomised trial. Lancet..

[6] Dondorp AM, Fanello CI, Hendriksen ICE, Gomes E, Seni A, Chhaganlal KD (2010). Artesunate versus quinine in the treatment of severe falciparum malaria in African children (AQUAMAT): an open-label, randomised trial. Lancet.

[7] World Health Organization (WHO). Artesunate for injection: adopted text for addition to The International Pharmacopoeia. 2011.

[8] Guilin pharma. WHO summary of product characteristics. 2011. p. 1–10.

[9] World Health Organization. WHO model prescribing information: drugs used in parasitic diseases. In: Transactions of the Royal Society of Tropical Medicine and Hygiene, vol. 85. 1991.

[10] Wilairatana P, Tangpukdee N, Krudsood S (2013). Tropical medicine & surgery practical aspects of artesunate administration in severe malaria treatment. Trop Mad Surg.

[11] Kadam P, Bhalerao S (2010). Sample size calculation. Int J Ayurveda Res..

[12] Mendes JR, Batista REA, Vancini-Campanharo CR, Lopes MCBT, Okuno MFP (2018). Types and frequency of errors in the preparation and administration of drugs. Einstein (São Paulo)..

[13] Reeves S, Pelone F, Harrison R, Goldman J, Zwarenstein M (2017). Interprofessional collaboration to improve professional practice and healthcare outcomes (Review). Cochrane Libr..

[14] Sari D, Şahin M, Yaşar E, Taşkiran N, Telli S (2017). Investigation of Turkish nurses frequency and knowledge of administration of intramuscular injections to the ventrogluteal site: results from questionnaires. Nurse Educ Today.

[15] Khan AN, Khan MU, Shoaib MH, Yousuf RI, Mir SA (2014). Practice nurses and pharmacists: a perspective on the expectation and experience of nurses for future collaboration. Oman Med J..

[16] Di Muzio M, De Vito C, Tartaglini D, Villari P (2017). Knowledge, behaviours, training and attitudes of nurses during preparation and administration of intravenous medications in intensive care units (ICU). A multicenter Italian study. Appl Nurs Res.

